# Current Advances and Evidence in Minimally Invasive Spine Surgery

**DOI:** 10.1155/2012/508415

**Published:** 2012-12-30

**Authors:** Richard G. Fessler, Zachary A. Smith, Nicholas Slimack, Justin S. Smith, Richard J. Parkinson

**Affiliations:** ^1^Department of Neurosurgery, Northwestern University, Chicago, IL 60601, USA; ^2^Department of Neurosurgery, University of Virginia, Charlottesville, VA 22908, USA; ^3^St Vincent's Clinic, Sydney, Darlinghurst, NSW 2010, Australia

It has been our privilege to serve as Guest Editors for this special focusing on the topic of current advances in *minimally invasive spine surgery*. Given the continuous and rapid advancement of spine technologies and techniques, an issue such as this can only begin by describing many of the advances in the field. However, with the critical help of our contributors, we hope this issue provides the readers with a better understanding of modern minimally invasive spine surgery.

The primary goal of minimally invasive surgery is to minimize procedure-related morbidity. Commonly, this is reflected in diminished tissue injury, less blood loss, shorter hospital stays, and decreased postoperative pain. To reflect this, two articles have discussed outcomes with two more established and commonly applied techniques. A. Wong and colleagues review the current literature reported on outcome data following the minimally invasive treatment of lumbar stenosis. Similarly, A. Habib and colleagues review outcomes following minimally invasive TLIF (transforaminal lumbar interbody fusion). 

Minimally invasive techniques were first applied to a more focused scope of surgical indications. However, more recent advances have allowed surgeons to use these techniques for more challenging pathologies. Consistent with this, a number of articles in this issue address the technical nuances of these advanced techniques. For instance, F. De Iure and colleagues describe their experience in 122 patients treated with percutaneous fixation for thoracolumbar burst fractures. M. Wang describes and presents outcomes of a less invasive technique for the percutaneous placement of iliac screws. Additional articles address such topics as advances in interspinous fixation, the use of fenestrated screw technology, and operative corridors for minimally invasive corpectomy. 

